# CT Appearance Pattern After Stereotactic Body Radiation Therapy Predicts Outcomes in Early-Stage Non-Small-Cell Lung Cancer

**DOI:** 10.3389/fonc.2021.746785

**Published:** 2021-10-11

**Authors:** Yan Yang, Gaohua Li, Shuyuan Li, Yuanhang Wang, Yanbo Zhao, Baiqiang Dong, Jin Wang, Ruiwu Zhu, Ming Chen

**Affiliations:** ^1^ Department of Radiation Oncology, General Hospital of Fushun Mining Bureau of Liaoning Health Industry Group, Fushun, China; ^2^ Department of Medical Oncology, General Hospital of Fushun Mining Bureau of Liaoning Health Industry Group, Fushun, China; ^3^ Department of Neurology, General Hospital of Fushun Mining Bureau of Liaoning Health Industry Group, Fushun, China; ^4^ Department of Radiation Oncology, Cancer Hospital of the University of Chinese Academy of Sciences (Zhejiang Cancer Hospital), Institute of Cancer and Basic Medicine (ICBM), Chinese Academy of Sciences, Hangzhou, China; ^5^ Department of Thoracic Surgery, General Hospital of Fushun Mining Bureau of Liaoning Health Industry Group, Fushun, China; ^6^ Department of Radiation Oncology, Sun Yat-sen University Cancer Centre, Guangzhou, China

**Keywords:** CT appearance pattern, lung cancer, prognostic prediction, SBRT, follow-up

## Abstract

**Backgrounds:**

Computed tomography (CT) appearance pattern after lung tumor stereotactic body radiation therapy(SBRT) might predicts survival. This study aimed to investigate the correlation between CT appearance pattern after SBRT and outcomes in patients with early-stage non-small-cell lung cancer (NSCLC).

**Methods:**

Clinical data of inoperable patients with early-stage NSCLC undergoing SBRT were retrospectively analyzed from 2012 to 2015 at the Zhejiang Cancer Hospital. The relationship between CT appearance pattern after SBRT and patient’s survival was analyzed.

**Results:**

The data from 173 patients with early-stage lung cancer treated with SBRT were analyzed. One month after SBRT, diffuse consolidation was seen in 17 patients, patchy consolidation in 28 patients, diffuse ground-glass opacity (GGO) in 10 patients, and patchy GGO in 22 patients. The survival time was significantly longer in the “no evidence of increased density” group compared with the “consolidation or GGO” group [2-year overall survival (OS) rate, 96.1% vs 89.3%; hazard ratio (HR), 0.36; 95% confidence interval (CI), 0.16–0.85; *P* = 0.015]. A similar trend was found in the progression-free survival (PFS) analysis (2-year PFS rate, 91.3% vs 85.0%; HR, 0.35; 95% CI, 0.13–0.95; *P* = 0.015) and distant metastasis free survival(DMFS) (2-year DMFS rate, 93.3% vs 87.1%; HR, 0.41; 95% CI, 0.20–0.86; *P* = 0.031). However, no significant difference was found in recurrence-free survival between the two groups (*P* = 0.212).

**Conclusions:**

One month after SBRT, the radiological change “no evidence of increased density” was prevalent. The OS, PFS, and DMFS were significantly longer in the “no evidence of increased density” group compared with the “consolidation or GGO” group. Further studies are needed to validate these findings.

## Introduction

Stereotactic body radiation therapy (SBRT) has been demonstrated as an effective critical alternative therapy for inoperable early-stage lung cancer and oligometastatic lung tumors (1–5). SBRT is characterized by high fractional dose and few treatment fractions. The accurate image guidance and delivery techniques restricted the high-dose radiation to the target areas, while doses outside the target areas declined rapidly to avoid damage to surrounding healthy tissue and critical organs (6–8).

SBRT has been reported to have a 5-year infield control of over 90% and a 5-year locoregional control of approximately 75% (9). Usually, elderly patients with inferior physical conditions are candidates for SBRT; once the disease is recurrent, the patients were usually unable to undergo salvage treatment. Besides recurrence, the post-SBRT lung injury after SBRT could have detrimental effects on outcomes in patients. Thus, distinguishing high-risk patients for local recurrence and radiation induced injury at follow-up is essential to make a personalized treatment plan and follow-up schedule.

Evolving radiological changes are common in 1–6 months after stereotactic ablative radiotherapy (SABR), and the typical patterns seen are subclassified into the following categories: diffuse consolidation, patchy consolidation, diffuse ground-glass opacity (GGO), and patchy GGO. Some radiological changes develop in HRFs, which are useful in suggesting local recurrence. Some characteristics do not significantly change, or exhibit a slow progression of fibrosis.

Many studies have investigated the relationship between radiological findings and recurrence. However, the sensitivity and specificity were not high enough to use CT appearance pattern as a predictor of recurrence. In addition, the pathophysiological changes leading to radiological findings after SBRT were unclear. One of the possible causes was that the probability of recurrence was low in these studies; therefore, analyzing the correlation between CT appearance pattern and survival outcome was difficult.

The present study assessed the relationship between CT imaging changes after SBRT and prognosis in patients with lung tumors who underwent SBRT. This study hypothesized that CT image information after SBRT was useful to predict outcomes in patients with lung tumors.

## Methods

### Patient Data

The present study was approved by the Ethics Committee and Institutional Review Board of General Hospital of Fushun Mining Bureau of Liaoning Health Industry Group (Committee’s Reference Number:20201201) and Zhejiang Cancer Hospital(IRB-2020-141 (Ke)). The medical records of patients with non-small-cell lung cancer (NSCLC) who underwent SBRT in Zhejiang Cancer Hospital from January 2012 to September 2015 were reviewed. Patients with early-stage (T1-T2N0M0) NSCLC lung cancer were selected in this retrospective study. NSCLC (including carcinoma, adenocarcinoma, large-cell carcinoma, and mixed-cell carcinoma) in all patients was confirmed by histology. However, patients with several tumors in the lungs were excluded from the analysis.

### Radiotherapy

The patients were immobilized by thermoplastic masks and vacuum cushion (Klarity Medical Products, NJ, USA) (10). The merit and demerit of two devices were introduced to the patients, and the patient choose one device with help and advices from technicians. Our routine method for arm immobilization was to let patient fold arms in a fixed posture with their hands across on the forehead, and then we took photos for marked points of the fixed posture. In the simulation, the free-breathing simulation CT was performed. The scan encompassed the upper margin of the second cervical spine up to the lower margin of the second lumbar spine with 3- to 5-mm-thick layers. The images were transferred to the treatment planning system (RayStation Launcher 4.5.1, RaySearch Laboratories AB, Sweden or Philips Pinnacle 9.2 treatment planning system, Amsterdam, The Netherlands) to delineate the tumor target area and the associated normal tissues. Overall, 10 series of CT images were reconstructed for each phase of respiration. Furthermore, the images were reviewed in the lung window and mediastinal window. The gross tumor volume (GTV) was manually contoured on each of the serial CT scans at one phase. The GTV inhale and GTV exhale were fused to generate the internal tumor volume (ITV). The planning target volume (PTV) expanded 5 mm in each direction based on the ITV. Moreover, organs at risk, such as bilateral lungs, spinal cord, trachea, bilateral chest wall, brachial plexus, heart, vessels, and esophagus, were contoured. The radiation dose limits to normal tissues were set according to RTOG0236. For large lesions (maximum cross-sectional diameter >4 cm) or adjacent vital organs, the dose in each fraction was reduced and the number of fractions increased. The prescribed iso-dose line percentage was 80% to cover 95% PTV and 100% to cover 100% ITV. The fraction dose was 7–12.5 Gy. Patients received 4–10 SBRT fractions (mean = 5) by 6 MV x-ray in 6–14 fields using the coplanar technique intensity-modulated radiation therapy (IMRT) or volumetric modulated arc therapy (VMAT) (ElektaSynergy, Stockholm, Sweden) or Varian Trilogy-SN5387 linear accelerator (Varian Medical Systems Inc., CA, USA) (11, 12). Cone-beam computed tomography (CBCT) scanning was conducted in a 360° standard rotation mode before each treatment.

### Follow-Up

Follow-up CT was conducted 1 month after completing SBRT, and thereafter every 3–6 months within 1 year and every 6–12 months after 1 year. Local recurrence was the recurrence occurring in the same lobe as the primary tumor, in the ipsilateral pulmonary, and hilar, mediastinal, and supraclavicular lymph nodes. Local recurrence was confirmed through follow-up CT according to RECIST and HRFs. FDG-PET/CT scans were recommended when local recurrence was suspected. In addition, centesis biopsy, continuing follow-up CT, or immediate salvage therapy was suggested by the physician if necessary.

### CT Image Database Assessment

The CT database of all patients treated with VMAT SABR for early-stage lung cancer was assessed. The post-SABR CT scans 1 month after SBRT were assessed in the radiological archives to identify representative acute radiological changes. All images and corresponding medical records of the selected cases were reviewed by an experienced radiation oncologist.

Follow-up CT scans were scored by two radiation oncologists (SY Li and Jin W). Image analysis was performed using RadiAnt DICOM Viewer (2020.2) for evaluating radiological changes in follow-up scans of patients with SABR. The scoring system for classifying acute radiological changes after SBRT of Max Dahele et al. was adopted to classify acute changes (11–13). Briefly, five kinds of acute changes were observed: diffuse consolidation, consolidation more than 5 cm in the largest dimension; patchy consolidation, consolidation less than 5 cm in the largest dimension; diffuse GGO, more than 5 cm of GGO; patchy GGO, less than 5 cm of GGO; and no evidence of increased density, no new abnormalities. This classification included patients with tumors that were stable, regressing, or resolved, or fibrosis in the position of the original tumor that was not larger than the original tumor. Any disagreement on the conclusion of the two evaluators was resolved by a discussion.

### Statistical Analysis

Multiple groups were compared by analysis of variance, while the pairwise comparison of the mean was conducted by the LSD test. The difference in the ratio was compared using the chi-square test. The log-rank test and Kaplan–Meier analysis were performed to explore the relationship between the CT pattern and recurrence probability in lung cancer after SBRT. It was considered to be statistically significant if bilateral *P <*0.05. All data were analyzed using SPSS (statistics version 21, SPSS Inc., IBM, IL, USA).

## Results

### Patient Characteristics

A total of 173 patients with early-stage lung cancer treated with SBRT were retrospectively analyzed. The median follow-up time was 28.3 months (range of 5.1–82.3 months). The median age was 76 years at the time of diagnosis, with 124 (71.3%) male patients and 49 (28.7%) female patients. Detailed baseline characteristics of the overall group, recurrence group, and nonrecurrence group are presented in **Table 1**. The 1-, 2-, and 3-year overall survival (OS) was 96%, 89%, and 79%, respectively; and the 1-, 2, and 3-year progression-free survival (PFS) was 88%, 80%, and 72%, respectively.

**Table 1:** Patient characteristics.

Characteristics	All patients (*n* = 173)
**Age (year), median (range)**	76 (43–88)
**Gender, *n* (%)**	
** Male**	124 (71.3)
** Female**	49 (28.7)
**KPS, median (range)**	90 (60–100)
**NCI Charlson score, median (range)**	1 (0–4)
**Tumor type, *n* (%)**	
** Central**	6 (3.5)
** Peripheral**	167 (96.5)
**Histology, *n* (%)**	
** Adenocarcinoma**	93 (53.8)
** Squamous cell carcinoma**	46 (26.6)
** NSCLC**–**NOS**	19 (11.0)
** SCLC**	2 (1.2)
** Others**	13 (7.5)
** FEV1, median (range)**	0.98 (0.44–3.39)
**Tumour diameter**	
** ≤1cm**	17 (9.8)
** 1-2cm**	71 (41.0)
** 2-3cm**	63 (36.4)
** 3-4cm**	21 12.1)
** 4-5cm**	1 (0.6)

FEV1, Forced expiratory volume in 1 second; KPS, Karnofsky performance status; NSCLC–NOS, non-small-cell lung cancer not otherwise specified; SCLC, small-cell lung cancer.

### Radiological Changes at 1 Month After SBRT

One month after SBRT, diffuse consolidation was seen in 17 cases, patchy consolidation in 28 cases, diffuse GGO in 10 cases, and patchy GGO in 22 cases. In 96 cases, no evidence of increased density was observed.

The patients were divided into two groups based on the radiological changes: the “no evidence of increased density” group and the “consolidation or GGO” group. No evidence of increased density were diagnosed in 13 of 17, 48 of 71, 32 of 63, 10 of 21 and 1 of 1 patients with a tumor diameter of ≤1cm, >2–3cm, >3-4cm and >4-5cm, respectively. No correlation between tumor size and CT appearance pattern after SBRT were found (p=0.096).

### Survival Difference

**Figure 1:**
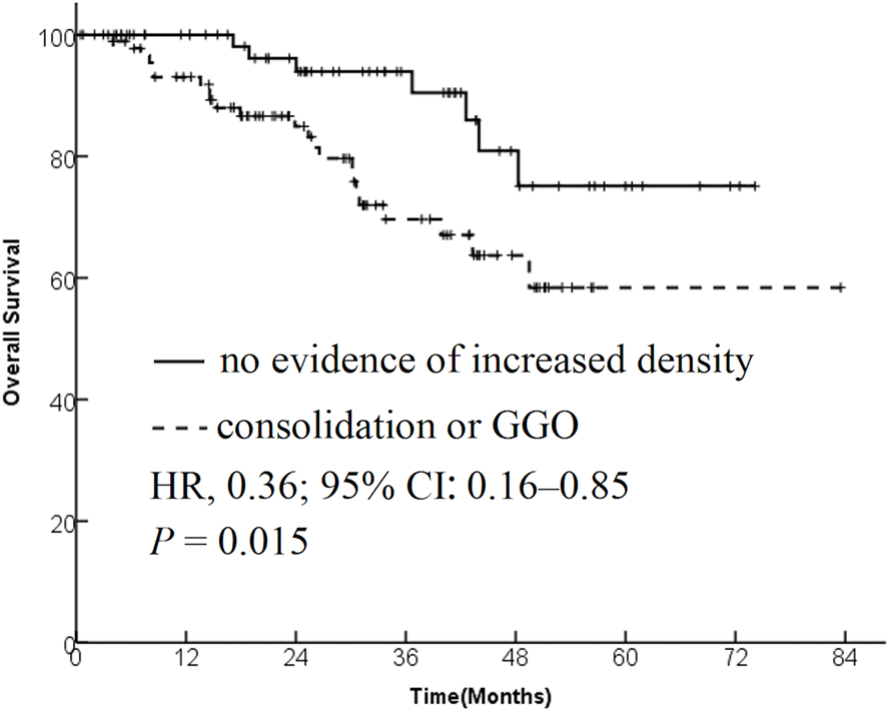
Kaplan–Meier curves for overall survival.

**Figure 2:**
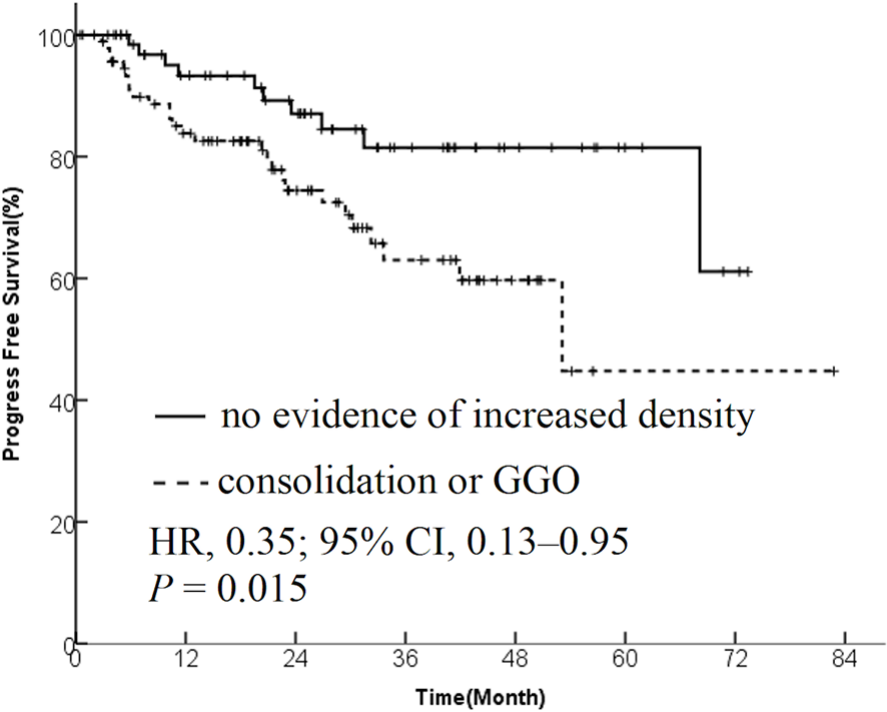
Kaplan–Meier curves for progression-free survival.

**Figure 3:**
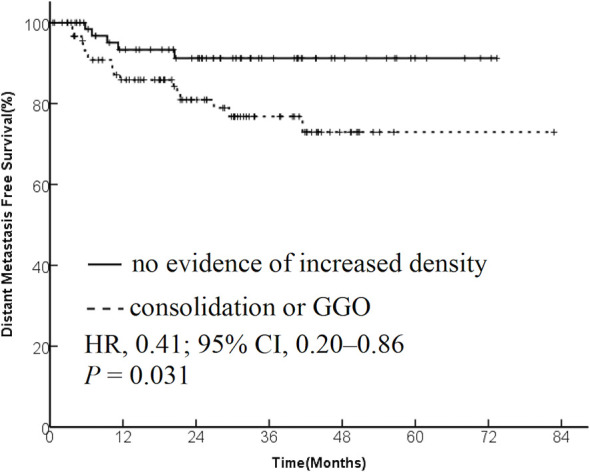
Kaplan–Meier curves for distant metastasis–free survival.

**Figure 4:**
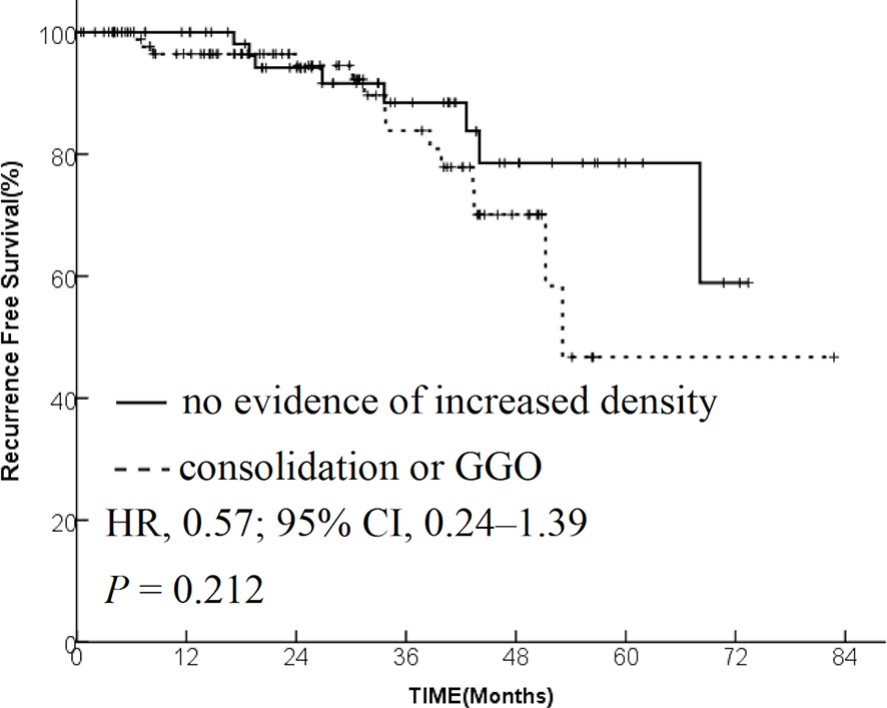
Kaplan–Meier curves for recurrence-free survival.

The patients were divided into two groups based on the radiological changes: the “no evidence of increased density” group and the “consolidation or GGO” group. Survival time in the “no evidence of increased density” group was significantly longer compared with the “consolidation or GGO” group (2-year OS rate, 96.1% vs 89.3%; HR, 0.36; 95% CI, 0.16–0.85; *P* = 0.015; **Figure 1**). A similar trend was found in PFS analysis (2-year PFS rate, 91.3% vs 85.0%; HR, 0.35; 95% CI, 0.13–0.95; *P* = 0.015; **Figure 2**) and DMFS (2-year DMFS rate, 93.3% vs 87.1%; HR, 0.41; 95% CI, 0.20–0.86; *P* = 0.031; **Figure 3**). However, no significant difference was found in recurrence-free survival between the two groups (2-year relapse free survival (RFS) rate, 94.2% vs 96.4%; HR, 0.57; 95% CI, 0.24–1.39; *P* = 0.212; **Figure 4**).

## Discussion

SBRT is characterized by accurate and high-dose radiation to tumor tissues as well as maximum protection of the surrounding normal tissues. These characteristics enabled SBRT to gain popularity. SBRT is expected to be one of the treatment alternatives to surgery for early-stage lung cancer.

Popularity of SBRT has led to an interest in radiological changes after SBRT. Although symptomatic Radiation pneumonitis (RP) after SABR is uncommon, radiographic radiation–induced lung injury (RILI) occurs frequently, as a result of ablative doses of radiotherapy delivered to the peritumoral region. In addition, accurately distinguishing recurrence from fibrosis is still an important clinical dilemma. Thus, elucidation of the clinical significance CT appearance pattern after SBRT seem to be particularly important, where salvage options, such as surgical resection, reirradiation, and combined chemoradiotherapy, are always not suitable because of poor PS status or high Charlson Comorbidity Index (CCI) score (14).

Radiological changes can be classified into five kinds in the acute setting (within 6 months of treatment). The degree of lung injury depends on multiple factors, including total dose and fractionation of irradiation, along with the target size. Radiological signs of fibrosis can potentially evolve even 2 years after treatment. Some radiological changes might develop into HRFs, which are correlated with recurrence (15).

This study revealed that radiological changes 1 month after SBRT predicted the prognosis. Moreover, patients with no radiological change showed a significantly longer survival compared with those who developed GGO or diffusion after SBRT. One of the possible reasons was that GGO or diffusion might develop into HRFs in the future. On the contrary, a large number of people who received SBRT died due to causes other than cancer. GGO or diffusion might also develop into RILI.

Another concern is that confounding factors might contribute to the presence of CT appearance after SBRT. Risk of radiographic pneumonitis or local recurrence would be correlated with tumor size and PTV volume. Furthermore, tumor size might be correlated with OS, PFS, and DMFS. Thus, the correlation between tumor size and CT appearance pattern after SBRT were analyzed, and no correlation was found between them. One possible reason is that the sensitivity to radiotherapy of an individual patient, other than tumor volume, is the decisive factor of CT appearance after SBRT. In addition, only patients with early stage (T1-2) were included in the study, and CT appearance did not differ significantly among them.

The systematic assessment of post-SABR CT images enables the prediction of prognosis. However, the sensitivity and specificity are not high enough. Radiomics is a newly developed technology that can extract innumerable high-dimension features from images and translate them into quantitative data (16) Previous studies have used radiomics to predict metastasis, OS, histologic subtype classification, distinction of malignant or benign lung nodules, and so forth. In the future, we plan to apply radiomics to improve the accuracy of prediction.

The CT findings after lung SBRT varied at different time point (17). It represented the different phase of radiation-induced lung injury. However, patients would like to have follow-up visits in local hospital in China because of some reasons. The follow-up CT scans did not always take place in our center. Only the CT scans at one month after SBRT were required in our hospital. The image data was completed in that time point. So in the study, we investigated the correlation between CT appearance one month after SBRT and outcomes.

In this study, the predictive value of radiological change 1 month after SBRT in patients with early-stage lung cancer was explored. However, this retrospective study also had some limitations. First, the follow-up CT scans were not periodic, and the database might not be comprehensive. Next, the patient’s number was rarely small. The results would need validation before applications for clinical use. Further research is needed in the future.

## Conclusions

One month after SBRT, the radiological change “no evidence of increased density” was very common. The OS, PFS, and DMFS in the “no evidence of increased density” group were significantly longer compared with the “consolidation or GGO” group. Further studies should be conducted to validate these findings.

## Data Availability Statement

The raw data supporting the conclusions of this article will be made available by the authors, without undue reservation.

## Ethics Statement

This study was approved by the Ethics Committee and Institutional Review Board of General Hospital of Fushun Mining Bureau of Liaoning Health Industry Group (Committee’s Reference Number:20201201) and Zhejiang Cancer Hospital (IRB-2020-141(Ke)). The patients/participants provided their written informed consent to participate in this study. Written informed consent was obtained from the individual(s) for the publication of any potentially identifiable images or data included in this article.

## Author Contributions

YY was responsible for project conceptualization, data collection and analysis, data interpretation, writing of the manuscript. GL and SL were responsible for project conceptualization, manuscript revisions, and editing of the manuscript. YW was responsible for statistical analysis. BD was responsible for patient data collection. JW and SL were responsible for score the imaging of follow-up CT scans. RZ was responsible for project conceptualization and editing of the manuscript and should be considered the guarantor for the article as a whole. MC was responsible for project conceptualization, and manuscript revision. All authors contributed to the article and approved the submitted version.

## Funding

This research has been supported by Liaoning Provincial Key Research and Development Program (Grant No. 2019JH2/10300024).

## Conflict of Interest

The authors declare that the research was conducted in the absence of any commercial or financial relationships that could be construed as a potential conflict of interest.

## Publisher’s Note

All claims expressed in this article are solely those of the authors and do not necessarily represent those of their affiliated organizations, or those of the publisher, the editors and the reviewers. Any product that may be evaluated in this article, or claim that may be made by its manufacturer, is not guaranteed or endorsed by the publisher.

## Abbreviations

4D-CT, Four-dimensional computed tomography; CBCT, cone-beam computed tomography; GTV, gross tumor volume; ITV, internal tumor volume; KPS, Karnofsky performance status; OAR, organs at risk; PTV, plan target volume; SBRT, stereotactic radiotherapy.
